# Accessing Numerical Energy Hessians with Graph Neural Network Potentials and Their Application in Heterogeneous Catalysis

**DOI:** 10.1021/acs.jpcc.4c07477

**Published:** 2025-02-10

**Authors:** Brook Wander, Joseph Musielewicz, Raffaele Cheula, John R. Kitchin

**Affiliations:** † Department of Chemical Engineering, Carnegie Mellon University, Pittsburgh, Pennsylvania 15213, United States; ‡ Department of Physics and Astronomy, Aarhus University, 8000 Aarhus, Denmark

## Abstract

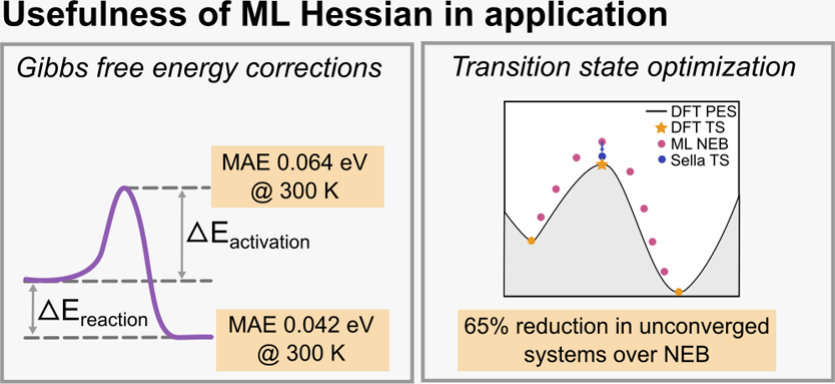

Access to the potential energy Hessian enables determination of the Gibbs free energy and certain approaches to transition state search and optimization. Here, we demonstrate that off-the-shelf pretrained Open Catalyst Project (OCP) machine learned potentials (MLPs) determine the Hessian with great success (58 cm^–1^ mean absolute error (MAE)) for intermediates adsorbed to heterogeneous catalyst surfaces. This enables the use of OCP models for the aforementioned applications. The top performing model, with a simple offset correction, gives good estimations of the vibrational entropy contribution to the Gibbs free energy with an MAE of 0.042 eV at 300 K. The ability to leverage models to capture the translational entropy was also explored. It was determined that 94% of randomly sampled systems had a translational entropy greater than 0.1 eV at 300 K. This underscores the need to go beyond the harmonic approximation to consider the entropy introduced by adsorbate translation, which increases with temperature. Lastly, we used MLP-determined Hessian information for transition state search and found we were able to reduce the number of unconverged systems by 65 to 93% overall convergence, improving on the baseline established by CatTSunami.

## Introduction

The potential energy Hessian captures the curvature of the potential energy surface (PES). The Hessian may be diagonalized to find the vibrational modes and frequencies of atomic species. Knowledge of the vibrational frequencies allows the vibrational contributions of the entropy to be estimated as well as the zero-point energy (ZPE). Knowledge of the ZPE and the entropy are necessary to calculate the free energy. When assessing whether a reaction is thermodynamically favorable, the free energy of reaction is considered. For constant pressure systems more particularly, the change in the Gibbs free energy is considered. To determine the rate of reaction, knowledge of the free energy of activation is necessary. In this sense, it is imperative to calculate the potential energy Hessian when seeking to answer the core questions of catalysis: the thermodynamic favorabilities and rates of reactions.

In heterogeneous catalysis, it is common to use the harmonic approximation, which assumes that the potential is harmonic about the minimum energy points. It is also common to assume the entropy is well treated by only considering the vibrational contribution to it, and that the vibrational energy from the surface may be neglected.^1−5^ These approximations neglect the translational, rotational, and electronic contributions to the entropy. The translational entropy is particularly important if the adsorbate is able to freely move about the surface. The harmonic approximation is poor at high temperatures where access to anharmonic energized states is possible and for more complex systems like those with solvent, zeolites, porous materials, and supported nanoparticles^6,7^ where other contributions also become important. There have been several proposed approaches to supplement the harmonic approximation. The hindered translator (and hindered rotor) model^8^ was introduced to account for the translational (and rotational) entropy of adsorbed species, obtained from partition functions associated with 1D surface diffusion and rotations hindered by activation energies. The complete potential energy sampling (CPES) method^9^ models instead the translational entropy from the explicit calculation of the 2D potential energy experienced by the molecules adsorbed to the surfaces, calculated with a series of constrained relaxations. Adsorbate entropies can also be estimated from the distribution of states extracted from molecular dynamics (MD) trajectories. In the quasi-harmonic approximation method,^10^ anharmonicity is accounted for by calculating the vibrational density of states using the velocities extracted from MD simulations. Anharmonic corrections can be computed also with the thermodynamic integration method, from the harmonic to the fully interacting system.^2^ Alternatively, adsorbate entropies can be obtained from enhanced sampling methods such as Metadynamics or the Blue-Moon approach.^7,11,12^ These approaches also enable the calculation of the entropy of transition states from MD.^13^

Since the release of Open Catalyst 2020 data set (OC20),^14^ there has been momentum in training novel machine learning (ML) model architectures^15−19^ to obtain versatile machine learned potentials (MLP)^20^ that may be used to accelerate or even replace density functional theory (DFT) in computational investigation of heterogeneous catalyst systems.^21,22^ In some cases, pretrained MLPs may benefit from additional fine-tuning on specific tasks. Fine-tuning is a training technique applied to pretrained models, which is commonly used in fields such as natural language processing to adapt neural network models to tasks outside the domain of the original pretraining data set.^23^ For molecular property prediction, fine-tuning has been shown to be effective both for improving the accuracy of previously learned properties for systems which are outside of the domain of the training set, and for learning to predict new properties while benefiting from useful latent information learned from the previous domain.^24−26^ Predicting the numerical Hessian of the potential energy as the Jacobian of the forces relies on MLP predictions of the forces, which pretrained OC20 MLPs were trained to predict. The potential energy Hessian is approximated numerically using finite differences. The displacements of atoms used in finite differences are at a length scale which are not necessarily represented in the OC20 data set, because the data set primarily contains relaxation trajectories of adsorbate catalyst systems.^14^ Therefore, it may be valuable to fine-tune pretrained MLPs on samples of the small displacements used to calculate Hessians numerically, so that the effect of these small displacements are adequately represented in the neural network.

**Figure 1:**
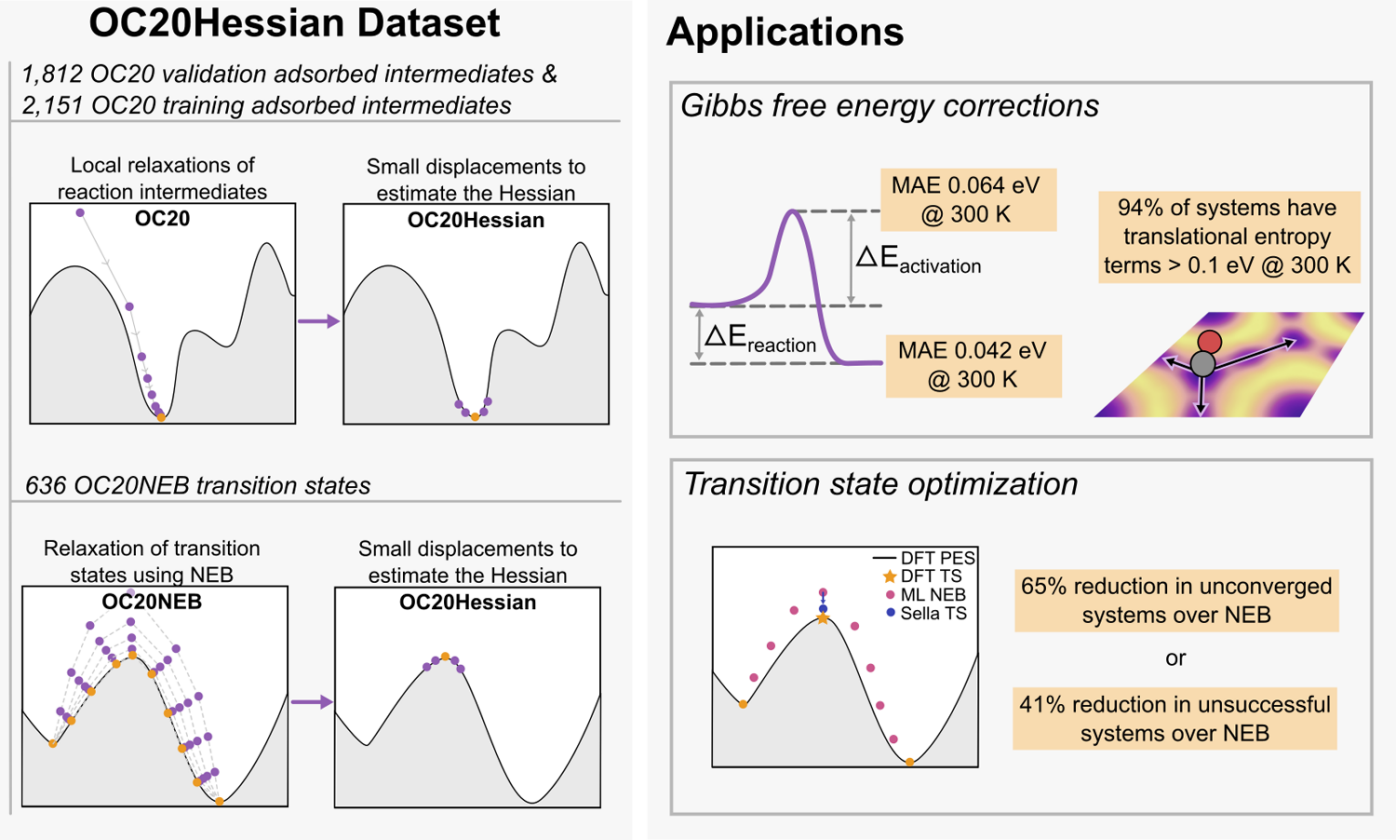
A summary of the work presented. We demonstrate that pretrained graph neural networks are able to determine the potential energy Hessian with high precision. This allows the Gibbs free energy corrections to be calculated with low errors. It is determined that many systems have significant translational entropy contributions at 300 K. The Hessians are also used for transition state optimization with Sella which greatly improves convergence.

Here, we seek to answer four outstanding questions about the use of OC20 trained MLPs, which are important to consider for practical use of the models in computational studies: (1) Can OC20 trained models be used to obtain Hessians accurately? (2) Can OC20 trained models be used to inexpensively augment entropy approximations? (3) How should Gibbs free energies most easily be accessed? (4) Can OC20 trained models be used to obtain energy Hessians that are useful in transition state optimization? The important components and outcomes of this inquiry are summarized in Figure 1.

To address these questions, we constructed a data set of 4599 numerical Hessians using DFT: 1812 randomly sampled relaxed structures from the OC20 validation data set, 2151 randomly sampled relaxed structures from OC20 training data set, and 636 transition states from the Open Catalyst 2020 NEB data set (OC20NEB).^22^ Using the calculated numerical Hessians, we assessed the performance of five pretrained models for determination of the numerical Hessian and found good agreement between DFT vibrational modes and those determined using the MLP. A mean absolute error (MAE) of 58 cm^–1^ was achieved by the top performing model on adsorbed intermediates. Using the ML and DFT vibrational modes, the Gibbs free energy corrections at 300 K were considered to ground the impact of ML errors on the resultant Gibbs free energy. For the top performing model, the MAE was determined to be 0.042 eV. This outperforms the commonly used approach of calculating the Gibbs correction for a particular adsorbate on one surface and assuming that value holds true for all surfaces, which has an MAE of 0.058. The opportunity of fine-tuning was also explored, which reduced the MAE further to 0.031 eV.

For 100 (of 1812) systems from the validation data set, the CPES^9^ approach was applied using MLPs, reducing entropy approximations to include the translational degrees of freedom for the adsorbed species. Across the 100 systems considered, the average difference in energy brought about by neglecting the translational degrees of freedom was 0.16 eV, with a maximum difference of 0.27 eV at 300 K. This underscores the value added by the low cost of the MLPs, which makes broader use of approaches which more completely treat the entropy feasible. Other techniques to go beyond the harmonic approximation such as ab initio molecular dynamics (AIMD)^27,28^ or enhanced sampling techniques could also be used with OC20 pretrained models, but consideration of these approaches was beyond the scope of this work.

To address the easiest method for accessing Gibbs free energy corrections, we considered the approach of using an ML potential to compute the Hessian. We also considered the simplest approach: constructing a lookup table with the average frequencies across the DFT data set. Although using the MLP performs the best (0.042 eV MAE at 300 K) for OC20 validation data, the lookup table is comparable (0.045 eV MAE at 300 K). The lookup approach does not extend to arbitrary adsorbates, which is especially important when transition states are considered. For this reason, we recommend using known corrections when possible and ML determined corrections where otherwise necessary.

Finally, we performed transition state optimization with Sella^29^ on the ML-determined transitions states from the OC20NEB^22^ data set using the numerical Hessian from the MLPs. Sella is an iterative molecular saddle point optimization algorithm that uses an updated approximate Hessian to follow the reaction coordinate. Providing the exact Hessian allows Sella to minimize the number of steps required to reach a saddle point within some threshold. We expect the Sella algorithm to be most effective for transition state optimization when the Hessian can be accurately and quickly determined. By performing Sella optimizations, we were able to increase the convergence rate from 80% to 93%. This demonstrates the usefulness of pretrained model Hessians on transition state optimization. It is likely the MLP could be applied to other Hessian driven transition state search algorithms such as the growing string method^30−32^ or earlier transition state search methods that rely on the Hessian.^33−38^ Success using graph neural networks (GNN) to perform transition state optimization with Sella has been successfully demonstrated for organic molecules with fine-tuning by Yuan et al.^39^

The contributions of this work are 5-fold: (1) creation of a data set of DFT 4599 numerical Hessians, (2) assessment of pretrained model performance at computing numerical Hessians, (3) fine-tuning of models to improve their performance for Hessian determination, (4) testing the ability to leverage MLPs to augment the harmonic approximation, and (5) Assessment of an OCP pretrained model for transition state optimization using Sella.

## Methods

### Generating the Data Set

1812 systems were sampled from the OC20^14^ validation data set. An additional 2151 were sampled from the OC20 training data set. The systems from the training set were limited to contain fewer than 100 atoms to reduce computational cost. Additionally, we considered all barriered reaction transition states presented in the OC20NEB^22^ data set. For all systems, a central finite differences approach without symmetry simplifications was used to determine the numerical Hessian. Calculations were performed using the Vienna Ab initio Simulation Package (VASP)^40−43^ with the revised Perdew–Burke–Ernzerhof (RPBE) functional.^44,45^ Two displacements of 0.0075 Å were performed for each direction. The electronic convergence criterion was met if the energy change between iterations was less than 1 × 10^–6^ eV. For the OC20 data set,^14^ the electronic convergence criterion was less strict (1 × 10^–4^ eV). The Hessians were also determined using an electronic convergence criterion of 1 × 10^–4^ eV for comparison with the 1 × 10^–6^ eV criterion. Only minute differences were observed between these convergence criteria across all OC20 validation and training systems, despite 1 × 10^–6^ eV being the recommended value. This alleviates any concern that the models would have issues with this task due to this discrepancy. Apart from this difference, all other settings were kept consistent with the OC20 data set. For more information about the calculation details and a histogram of the absolute error associated with the different electronic convergence criteria, see the Supporting Information. The data set and associated code are available on GitHub at https://github.com/jmusiel/gibby/tree/main.

### Calculating the Gibbs Energy Corrections

The Gibbs free energy corrections (*G*_corr_) have three terms which depend on the vibrational modes: the zero-point energy (ZPE), vibrational specific heat (*C*_p_) term, and the entropy term (*TS*) as shown in eq 1. The specific heat term (eq 3) may be neglected because an equal and opposite term appears in the entropy (eq 4). In eqs 2–4, *h* is Planck’s constant, *k* is the Boltzmann constant, and *T* is the temperature. For this work, we used the HarmonicThermo class in the Atomic Simulation Environment (ASE)^46^ to calculate the Gibbs corrections. If an imaginary vibrational mode was found, it was discarded for the assessment of the Gibbs energy. Imaginary vibrational modes could be zero-valued but instead correspond to small negative eigenvalues because the central finite-difference approach approximates the curvature insufficiently. If zero-valued, these should be interpreted as a loss of a vibrational degree of freedom in favor of a translational mode. In this case, to get an accurate entropy, the 2D translational entropy should be calculated. If the actual value is small and real valued, the vibrational mode should be interpreted as a frustrated vibration. In this case, the finite difference approach should be altered to better capture the curvature correctly. These modes usually do not contribute significantly to the enthalpy, but they do contribute substantially to the entropy.

For evaluation of the performance of ML models in comparison to explicit calculation we consider two cases (1) mean per adsorbate DFT and (2) Adopt-a-value DFT. This was done to develop two additional useful baselines. Because the Gibbs corrections for the same adsorbate on different surfaces are often similar, it is reasonable to use information about the correction on other materials/surfaces to estimate the correction for a new surface. This is what these baselines are meant to capture. For mean per adsorbate, the mean ordered frequencies across all systems are computed per adsorbate. Then the error between the Gibbs energy corrections obtained using the actual frequency values and the mean frequency values is computed for every system. For Adopt-a-value, the systems are iteratively considered and exhaustively paired with other systems with the same adsorbate on different surfaces. For each pair, the difference in energy is the error. The error is averaged across all systems. This case is the most similar to the common practice^1,4,5^ of calculating the per adsorbate Gibbs corrections on one surface and taking them to be true across all surfaces.
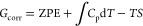
1
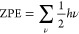
2
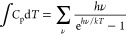
3
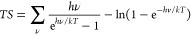
4

### Hessian Accuracy Metrics

The Hessian matrix of the potential energy was predicted for each system in the data set using a variety of GNN MLPs with high performance on OC20 benchmarks. A numerical central finite differences approach was used to obtain the Hessian matrix from the MLP, which is the same approach that is used by VASP. Since VASP is not analytically differentiable, the data we use as ground truth comes from the central finite difference method on VASP. The numerical approach was chosen for the MLP over an automatic differentiation approach after testing showed that automatic differentiation was slower than the finite differences approach for the GNNs we tested. Using central finite differences on the MLP was also found to more closely match the results of VASP central finite differences. More information on the choice of finite differentiation instead of automatic differentiation can be found in the Supporting Information.

We examined three different metrics for assessing the accuracy of Hessian matrix predictions. First, we are interested in the overall accuracy of the Hessian prediction itself, as a way of examining the overall efficacy of the MLP. The MLPs have been trained on force and energy property labels, but here they are used to predict the curvature which is the second derivative of energy, or the first derivative of the forces. To assess the overall Hessian accuracy, we look at the MAE of the sorted frequencies. Alternatively, we could use the sorted eigenvalues, but these scale with the square of the frequencies, and therefore capture the same information. Second, we are interested in the performance of the Hessian as a tool for predicting the Gibbs free energy correction, which is a function of the frequencies. Therefore, we use the MAE of MLP Hessian predicted Gibbs free energies versus VASP Hessian predicted Gibbs free energies. This metric should generally correspond to the overall accuracy of the Hessian, but may be biased by significant errors in the very high magnitude and very low magnitude frequency ranges. Third, we are interested in the use of the Hessian to identify transition states versus relaxed states. Ideally, transition states are characterized by a single imaginary frequency, and therefore the only frequency of interest is the largest imaginary where applicable otherwise smallest real (LI/SR) frequency. We measured the MAE of this LI/SR frequency to examine the effectiveness of the Hessian prediction for identifying, and optimizing toward, transition states.

### Fine-Tuning Experiments

To examine the effects of fine-tuning a pretrained Equiformer V2 checkpoint, we used data generated by vibration calculations on local minima from the OC20 training data set. We selected the 153 M parameter Equiformer V2 checkpoint for the starting model weights because it is the best performing pretrained checkpoint we tested. Instead of training the model to directly predict the Hessian, we fine-tuned it only to predict the same force and energy properties it was already trained for, with a different learning rate on a different data set.

The difference between the fine-tuning step and the pretraining is the data set used for fine-tuning, which contains numerous single point calculations generated by DFT using the central finite differences approach to generate the Hessian. These single point calculations contain numerous small displacements of the adsorbate atoms, smaller than would typically be found in a relaxation trajectory which composes the original pretraining data set. To select a learning rate, we performed hyperparameter optimization and determined a learning rate of 5 × 10^–6^ to be best. We validated the model’s force and energy performance on a held-out set of relaxed structures to determine when to stop training. Using this held-out set, we determined the optimal stopping point to be 12 epochs, but we also measured the accuracy on our test sets after each epoch from 0 to 20 to examine the performance trends associated with fine-tuning.

We measured the fine-tuned model’s performance in predicting the Hessian and Gibbs correction on two test sets comprised of reaction intermediates and transition states. To assess the Hessian prediction performance we used the MAE (ML versus DFT) of the LI/SR frequency. The LI/SR frequency is expected to be indicative of the model’s ability to accurately optimize transition states using the diagonalized Hessian. We also computed the overall Gibbs energy correction error by taking the MAE (using the ML versus the DFT Hessian) of the Gibbs energy correction.

Because we fine-tuned the model only on relaxed structures, we expect there to be poorer performance for transition state structures. In particular, we expect to see a significant difference in the performance of the model on the LI/SR frequency, which corresponds to the reactive mode. Therefore, we specifically examined the distribution of errors on just the LI/SR frequencies, comparing the distribution of the relaxed structures from the OC20 validation set to the saddle points from the OC20NEB transition states set.

### Complete Potential Energy Sampling

To facilitate the evaluation of the translational entropy, we created a suite of tools to perform potential energy sampling by the procedure described by Jo̷rgensen et al.^9^ These tools are available on GitHub at https://github.com/jmusiel/gibby/tree/main. The tool takes as input the adsorbate, slab, and an ASE^46^ calculator object to perform constrained relaxations of the adsorbate on the slab. There is an ASE calculator object compatible with evaluating OC20 pretrained models, making their use facile. The constrained relaxations are performed on a grid with adjustable spacing. Results are cached so they may be accessed at a later time. Once the constrained relaxations are performed, a surrogate PES is constructed and used to evaluate the translational partition function and entropy by eqs 5 and 6, which was taken from the work by Jo̷rgensen et al.^9^ We also created classes similar to the ASE HarmonicThermo class, to allow for the rapid evaluation of the Gibbs free energy for a constructed PES and vibrational modes.
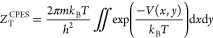
5
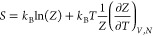
6

To validate the CPES approach, 100 systems from the 1812 OC20 validation set were analyzed. For each system, a mesh grid of points was constructed across the unit cell surface with a spacing of 0.5 Å. For each one of these points a constrained relaxation of the adsorbate was performed using the 31 M parameter Equiformer v2^19^ pretrained model. The adsorbate was prevented from dissociating using Hookean constraints which allowed bond elongation of 1.3× the relaxed bond length, beyond which a 10 eV/Å force was applied. Additionally, all surface atoms were fixed and the adsorbate center of mass was fixed in the directions parallel to the surface, but allowed to move in the direction normal to the surface. Using the relaxed energies across the meshgrid, a finer surrogate grid was constructed using cubic spline interpolation. The surrogate mesh grid was used to integrate the potential energy across the surface to determine the associated partition function by eq 5. With the partition function, the entropy may be readily assessed (eq 6). DFT single points were also performed on the ML relaxed systems. These single points were performed with the functional and settings consistent with the OC20 data set.^14^

### Transition State Optimization with Sella

Transition state optimization algorithms such as Sella make use of an exact or approximate Hessian matrix to efficiently drive the optimization toward the nearest saddle point.^29^ We measure the effectiveness of Hessian matrix calculations from models trained on OC20 for performing transition state optimization by examining the effects of using Sella to optimize the transition states identified in the OC20NEB data set. From our observations, Sella is sensitive to hyperparameter initialization, so we performed a hyperparameter optimization to determine the appropriate parameters to be used. The parameters selected maximized the convergence rate over 500 steps for the 153 M parameter Equiformer V2 model on the OC20NEB transition state data set. The convergence criterion (γ) was set to 0 to ensure that the Hessian matrix was recalculated and used at every step in the optimization. A trust radius of η = 7 × 10^–4^ Å was used. More details on the specifics of all the hyperparameters used for the Sella optimization can be found in the SI.

Sella’s performance as a transition state optimizer is compared to the CatTSunami baseline which identified transition states using the nudged elastic band (NEB) technique. For the Sella approach, the ML NEB optimized transition state structure is refined with Sella to convergence, or for 500 steps, whichever happens first. We compare two variants of this approach: (1) unconverged structures from the NEB approach or from Sella are removed completely to improve accuracy, and (2) where Sella converged structures are prioritized but NEB converged structures are used in the event that Sella fails to converge to improve convergence rate. We track several metrics to assess the performance of the transition state optimization: convergence rate, maximum force magnitude (Fmax), activation energy (*E*_A_) error, success rate when converged, and success rate overall. The convergence rate is the percentage of systems which reach a transition state below Fmax criteria (0.01 eV/Å for Sella, 0.05 eV/Å for NEBs). The Fmax is the magnitude of the maximum force when a DFT single point is performed on the ML-relaxed transition state. The *E*_A_ energy residuals are the differences between the activation energy of the DFT single point performed on the ML optimized transition state, and the result of a DFT NEB calculation starting from the same system. The success rate when converged is the proportion of converged systems where the activation energy of the DFT single point performed on the ML optimized transition state is accurate to within 0.1 eV of the energy given by the DFT-optimized transition state from an NEB calculation. Lastly, the overall success rate is the proportion of all systems that are both converged and within 0.1 eV of the energy given by the DFT NEB.

## Results and Discussion

### Hessian Accuracy from Pretrained Force Models

**Figure 2:**
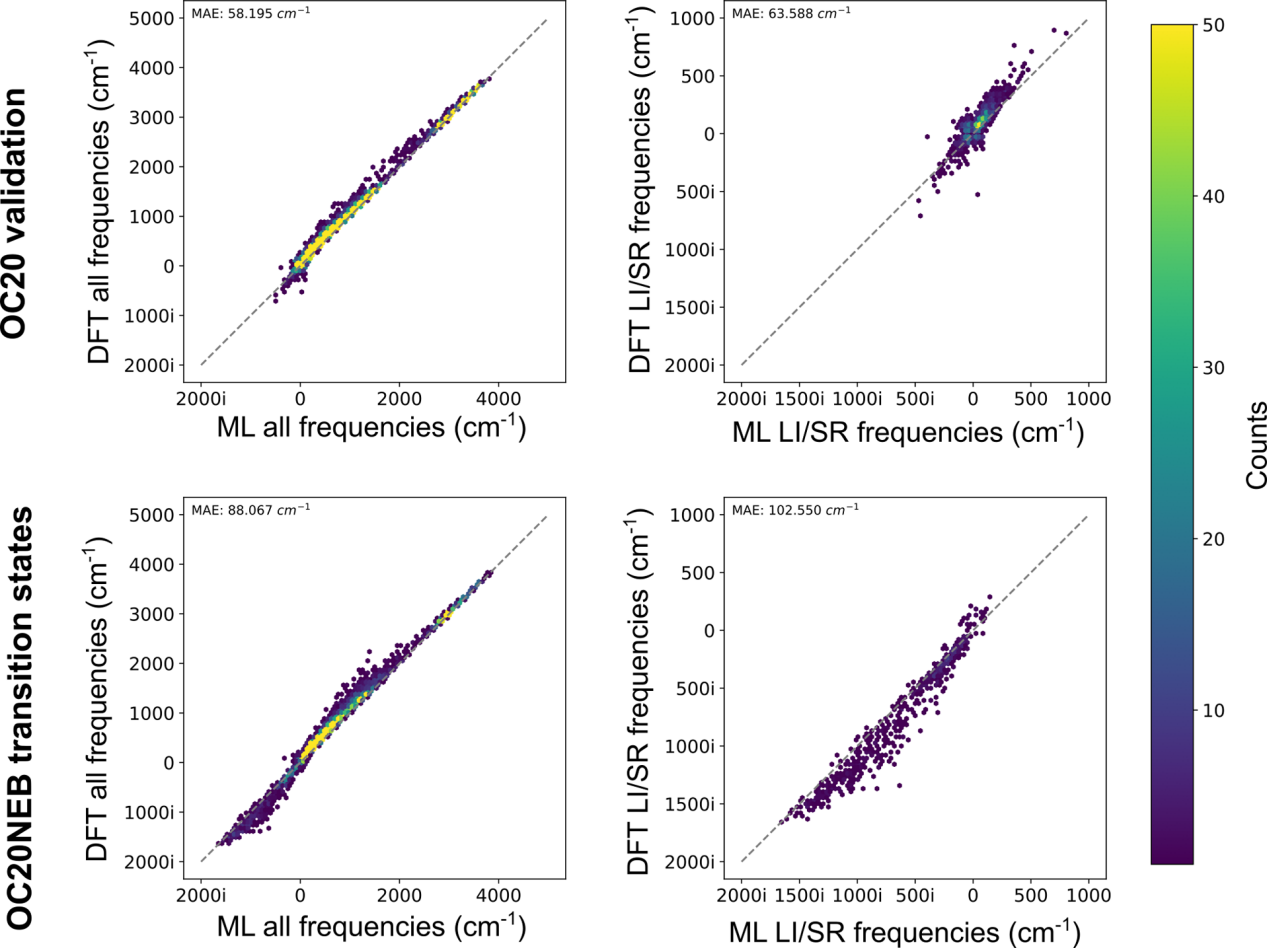
Parity plots of the frequencies for all vibration modes and the LI/SR frequencies of the Hessian predicted by VASP versus the Hessian predicted by the 153 M parameter Equiformer V2 via finite differences.

We measure the accuracy of predicting the Hessian from a pretrained GNN by numerically computing the gradients of the forces via central finite differences. This approach is identical to the one implemented in VASP, which was used to calculate the DFT numerical Hessians. The performance of the top performing model (Equiformer V2 153 M parameters) is shown in Figure 2 on the OC20 validation systems (top) and the OC20NEB transition states (bottom). For both data types, a parity plot of the vibrational frequencies (left) reveals the high degree of parity between the DFT and ML results. There is, however, a small systematic bias. The models systematically predict larger magnitude eigenvalues and therefore the vibrational frequencies tend to be slightly larger than the actual values. Interestingly, this is the opposite effect to that noted by Deng et al.^47^ for three different MLPs on out of domain systems. This bias can and will be corrected for in the application of the Hessian. Special attention is also paid to the LI/SR vibrational frequency (shown on the right). In the case of a transition state, this should correspond to the eigenmode of reaction. For the transition state systems, the distribution of the LI/SR frequency is shifted to the left when compared to the OC20 validation systems as we would expect. For nonreacting systems, there are still some with imaginary eigenmodes. In this case, it is likely the curvature is shallow and the imaginary frequency is an artifact of the numerical technique. Still, for this important frequency on the OC20NEB transition states, we find there is good parity between the ML and DFT values. This is a promising signal for the use of OC20 pretrained MLPs for transition state optimizations which rely on the Hessian.

### Fine-Tuning a GNN on Vibration Single Points

**Figure 3:**
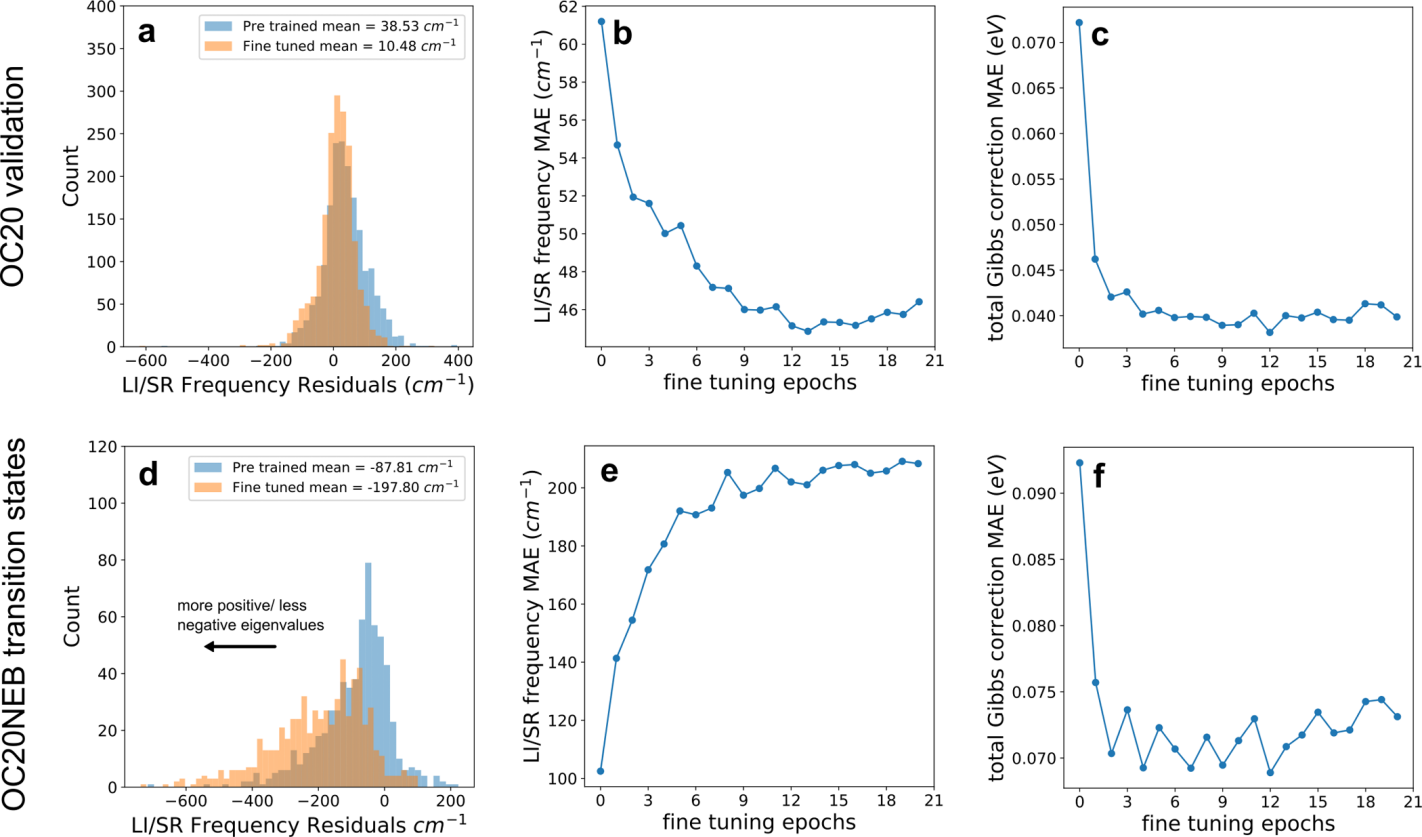
Impact of fine-tuning for the OC20 validation (top) and OC20NEB transition states (bottom). (a and d) A residual plot of the LI/SR frequency for the best performing fine-tuned checkpoint–trained for 12 epochs, (b and e) the evolution of the MAE in the LI/SR frequency with number of training epochs, and (c and f) the evolution in the total Gibbs correction MAE at 300 K with the number of training epochs.

To determine the impact of fine-tuning on model performance for the auxiliary task of determining the numerical Hessian, we fine-tune the model on Hessian single points sampled from local minimum states on the PES. Systems used for fine-tuning were sampled from the OC20 training data set; transition states are not represented in the data. For each adsorbate-slab system, two small displacements are included in each direction for every adsorbate atom. Figure 3 shows that fine-tuning improves the accuracy of predictions of the LI/SR frequencies (Figure 3b) and the Gibbs free energy corrections for adsorbed intermediates (from OC20 validation) (Figure 3c). Fine-tuning also improves the Gibbs free energy prediction accuracy for transition state calculations (OC20NEB) (Figure 3f), but it degrades the prediction of the LI/SR frequencies (Figure 3e). This is sensible since the training data was comprised of nonreactive systems which should not have strong, negative eigenvalues like those of the LI/SR frequency of reactive systems.

To test whether training on local minimums explicitly biases the model toward noncomplex frequencies, we examine the distribution of residuals for the LI/SR transition state frequency between the pretrained and fine-tuned models (after 12 epochs) in Figure 3a,d. We observe a shift toward a more normal distribution of residuals in the OC20 validation calculations (Figure 3a), while also observing a shift toward a more negatively skewed distribution of residuals for the transition state calculations (Figure 3d). The shift toward negative residuals shows that the lowest frequency predicted by the fine-tuned model is more often erroneously high, shifting toward noncomplex frequencies, which would be observed in the training set. This means that fine-tuning the model on displacements sampled about local minima decreased its propensity to predict imaginary vibrational modes, while increasing its overall predictive accuracy. Therefore, we conclude fine-tuning on small displacement data around local minima to be an effective strategy for improving the model’s Hessian predictions in general, but not for improving its capacity to identify optimal transition states. This fine-tuning behavior is useful for cases like predicting Gibbs free energy corrections for a wide variety of data sets, including transition state data, where the entire Hessian is used. However, if the Hessian prediction is used for identifying transition states, or transition state optimization strategies, then the training data selected for fine-tuning should be representative of transition state calculations to provide any improvement.

### Gibbs Free Energy Corrections

Using the potential energy numerical Hessians from DFT and a variety of machine learning models, the Gibbs free energy errors are calculated and compared at 300 K in Table 1 for the OC20 validation systems (top) and the OC20NEB transition states (bottom). For the OC20 validation systems, ML computed approaches may be compared to Adopt-a-value DFT, which is similar to the approach that is commonly taken by researchers whereby the correction is calculated using DFT for each adsorbate of interest in a single surface, and then that value is assumed to hold for all surfaces. The Equiformer V2 153 M (EqV2 153M) parameter model with a bias correction of 0.240 eV/Å^2^ outperforms the Adopt-a-value approach which have MAEs of 0.042 and 0.058 eV, respectively. The average time required to compute the Hessian matrix for the OC20 validation systems was 2454 GPU seconds per system using DFT with finite differences, and 4.7 GPU seconds per system using EqV2 153 M with finite differences. As expected, computing the Hessian matrix with ML is approximately 2 orders of magnitude faster than running the same computations with DFT. Notably, the ML approach could be further accelerated by batching more than one perturbation on the GPU at once, which was not implemented for this work.

**Table 1:** Mean Absolute Errors of Gibbs Energy Correction Terms^a^

local minimum correction MAE			
method	ZPE [eV]	*C*_p_-TS [eV]	total Gibbs [eV]
fine tuned EQ2	0.017	0.019	0.031
EQ2 153 M corrected	0.025	0.023	0.042
mean per adsorbate	0.029	0.023	0.045
adopt-a-value	0.042	0.024	0.058
EQ2 153M	0.042	0.027	0.062
EQ2 31M	0.059	0.031	0.080
GemNet T	0.074	0.036	0.101
PaiNN	0.121	0.044	0.151
SchNet	0.147	0.058	0.201

**transition state correction MAE**			
method	ZPE [eV]	*C*_p_-TS [eV]	total Gibbs [eV]
EQ2 153 M corrected	0.046	0.020	0.064
fine tuned EQ2	0.047	0.022	0.068
EQ2 153M	0.066	0.028	0.092
GemNet T	0.071	0.032	0.102
EQ2 31M	0.098	0.042	0.139
SchNet	0.108	0.039	0.143
PaiNN	0.158	0.062	0.217

a Each correction term contributes to the total Gibbs energy correction. Predictions made on local minimum relaxations and transition state calculations taken from the OC20 validation set. The methods column corresponds to the MLPs used to perform a vibration calculation on each system in the data set, except for the mean per adsorbate approach, which uses the average correction over each adsorbate and only applies to local minimum calculations.

It can be observed that the performance on this auxiliary task is correlated with the OC20 training metrics. EqV2 153 M model has the lowest MAEs for force and energy on OC20 and the remaining ordering is retained. This is somewhat true for the transition states as well, although PaiNN and GemNet T are incorrectly ordered. Across the models, performance is poorer on the transition states than on the OC20 validation systems except SchNet. Poorer performance is expected given that transition states do not appear in the OC20 training data.

Given the functional forms of the ZPE and entropy terms in eqs 2 and 4, the entropy term is more sensitive to relative errors in smaller frequencies and the ZPE is more sensitive to relative errors in larger frequencies. For both Adopt-a-value and the EqV2 153 M model, the error contributions for these two terms are roughly equal for the OC20 validation set. There is a marked improvement in the MAE with fine-tuning (fine-tuned EQ2) giving an overall MAE of 0.031 eV for OC20 validation and 0.068 eV for OC20NEB transition states, which is the lowest of all. The percent improvement with fine-tuning compared to the pretrained EqV2 153 M model is larger for the ZPE term than the entropy term, indicating that the improvement is not as strong for smaller frequencies.

For the OC20 validation systems, we also present the mean per adsorbate approach. This gives an improvement over Adopt-a-value (0.045 eV versus 0.058 eV MAE), but is still bested by the corrected EqV2 153 M model. The mean ordered vibrational energies for each adsorbate are included in a table in the Supporting Information, so that they may be used. The distribution width varies by adsorbate. Sensibly it is roughly correlated with the number of atoms in the adsorbate (larger adsorbates tend to have wider distributions of corrections than smaller adsorbates). A box and whisker plot of the adsorbate Gibbs corrections at 300 K has been included in the Supporting Information as well.

### Complete Potential Energy Sampling

**Figure 4:**
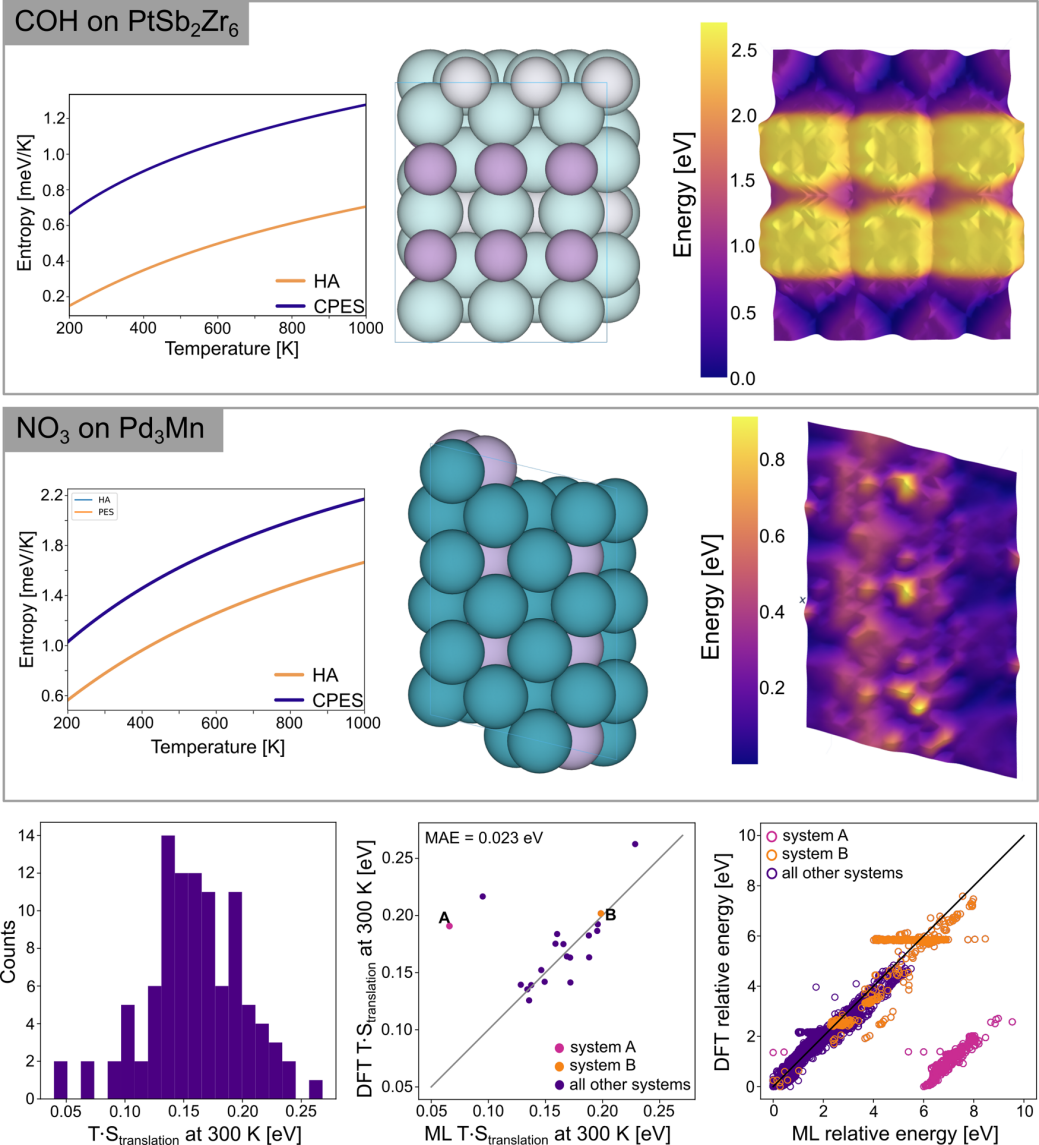
Two examples of the complete potential energy sampling (CPES) technique for COH on an Pt–Sb–Zr alloy and NO_3_ on an Pd–Mn alloy. For each, the slab (center), potential energy surface (right), and the entropy profiles versus the temperature is shown for the harmonic approximation (HA) and CPES (left). The distribution of the translational component to the entropy correction evaluated at 300 K across 100 randomly sampled systems from validation (bottom, left). The parity between ML determined and DFT single point validated translational entropy Gibbs corrections (bottom, center). The parity between the relative adsorption energies used to construct the PES from ML and DFT single points (bottom, right). In the bottom, center and bottom, right figures two systems (A and B) have been highlighted so the impact of outliers in the relative energies (right) on the translational entropy term (center) can be seen.

We use the Equiformer V2 31 M parameter model to perform complete potential energy sampling (CPES) to obtain a more complete entropy correction term of the Gibbs free energy for 100 systems from the 1812 OC20 validation set. A summary of the results from this analysis is shown in Figure 4. For two example systems, the temperature profile of the entropy using the harmonic approximation and adding the CPES translational entropy are shown (left), along with an image of the slab (center), and the potential energy surface (PES). For both examples, there are large swaths of accessible (low change in energy) states, which can be seen in the PES. For the top example, the Sb (purple) atoms are unfavorable for COH to bind to, which gives the high energy (yellow) regions on the PES.

Across all 100 systems, the average difference in the correction with CPES was 0.16 eV at 300 K, with a maximum of 0.27 eV. The distribution of all differences in the correction are shown in the lower left of Figure 4. There were some additional issues with errors in the models because of the Hookean and fixed center of mass constraints. This resulted in a loss of density for the meshgrid, which for most adsorbate–surface combinations was modest (4% of placements on average), but in some cases caused the approach to be unusable (a maximum of 48% was observed). For adsorbates that are stable in the gas phase, there were some adsorbate–surface combinations where a significant number of adsorbate placements desorbed over relaxation. This undermines the fidelity of the approach which is intended to capture 2D translational freedom, not 3D.

For a subset of 17 systems which did not have a significant proportion of desorbed or errant systems, DFT single points were performed on the ML relaxed structures. A comparison of the relative DFT single point energies (relative to the minimum) and the relative ML energies can be seen in the lower, right of Figure 4. The majority of systems have good energetic parity, but there are a few exceptions. For system B (orange), there are a large number of points which are determined to be equal in energy by DFT, but ML erroneously finds the energies to have a distribution of values. For system A, which is shown in pink, the minimum energy is erroneously small and far away from the distribution. This is the reason that the primary cluster of data is shifted away from the parity line but still correlated. The impact these artifacts have on the translational entropy component to the Gibbs free energy is shown in the bottom, center of Figure 4. The differences with system B have a minute impact on the overall correction, while the differences with system A have a large impact. Similar artifacts can be seen for the OC20Dense data set^21^ for the GemNet-OC model,^18^ and a figure supporting this has been included in the Supporting Information. There are two large outliers when comparing the translational entropy terms by ML and supplemented with DFT single points. Both of these were caused by the same issue described for system A: having a few ML energies that are far away from the energy distribution. The other outlier can not be seen the bottom, right of Figure 4 because the overall magnitude of its energy distribution is small so it is masked by the noise in other systems. In these cases, the translational entropy has been underestimated by ML. Still, the MAE in the translational entropy term by ML with reference to the DFT single points is just 0.023 eV at 300 K.

The results from Jo̷rgensen et al.^9^ for CO and O on the Pt (111) surface were reproduced with ML and DFT relaxations. A comparison of the approaches reveals very good agreement and a figure supporting this has been included in the Supporting Information.

Overall there is good agreement between the DFT and ML PES and Gibbs corrections, but the outliers in the bottom, center figure suggest that supplementing with DFT single points can be advantageous. These results underscore the importance of considering translational degrees of freedom for the entropy in adsorbed systems. The translational entropy correction will be even more impactful at higher temperatures and therefore should not be ignored. The MLPs perform well for this task, which would be very expensive to perform with DFT.

### Transition State Search with GNNs

We use Sella to perform transition state optimization on the ML-relaxed transition states from the OC20NEB^22^ data set. Table 2 shows that depending how it is applied, Sella may be used to improve either the success or convergence rate depending on the user’s priority for these outcomes. There are two defined success rates: the success when converged and the overall success. The success when converged is the success over the subset of calculations where the algorithm converged, while the overall success rate includes unconverged calculations explicitly as failures. Selecting only systems where both the NEB algorithm and the subsequent Sella algorithm converged gives several benefits: more accurate energies, lower forces, and a higher success rate are achieved across each of the reaction types. 95% success can be seen as the maximum success rate because this is the success rate obtained in the CatTSunami baseline when ML is used to preoptimize the NEB, followed by a DFT NEB. Despite the NEB being constrained about the reactant and product configurations, in 5% of cases the DFT NEB following ML prerelaxation found a transition state structure that was more than 0.1 eV lower in energy. These improvements come with the cost of accepting a smaller proportion of systems (9.6% fewer converged for all reactions). This is a fairly significant trade-off when converging as many systems as possible is a priority, but it does provide higher confidence in the accuracy of the calculations which are accepted. Alternatively the Sella refinement can be used on all systems, and any systems where either the NEB or Sella algorithm converges are accepted to improve overall convergence (93% for all reactions) when compared to using the NEB alone (80.2% converged for all reactions). In this case, the proportion of systems that converge is greatly improved compared to the CatTSunami baseline, while the energy MAE is only marginally degraded. Because this approach improves convergence, the overall success, which includes unconverged calculations as failures improves greatly for dissociations and transfers. The maximum force also improves, indicating that the Sella algorithm may be more successful than the NEB algorithm in finding valid relaxed saddle points with the MLP according to DFT.

**Table 2:** A Comparison of Key Metrics for the CatTSunami Baseline and Two Applications of Sella for Transition State Optimization^a^

method	mean fmax [eV/Ang]	energy MAE [eV]	converged [%]	success when converged [%]	success overall [%]
All Reactions					
CatTSunami	0.159 (0.078)	0.060 (0.149)	80.15	91.54	73.37
Sella refined (any)	0.137 (0.074)	0.069 (0.186)	92.99	89.79	83.50
Sella refined (both)	0.132 (0.073)	0.041 (0.113)	70.60	95.20	67.21
Desorptions					
CatTSunami	0.135 (0.077)	0.027 (0.031)	97.23	98.78	96.05
Sella refined (any)	0.098 (0.078)	0.035 (0.058)	97.66	98.40	96.09
Sella refined (both)	0.095 (0.075)	0.029 (0.063)	95.26	99.59	94.86
Dissociations					
CatTSunami	0.164 (0.085)	0.039 (0.106)	79.66	90.43	72.03
Sella refined (any)	0.147 (0.065)	0.042 (0.130)	97.48	88.56	86.33
Sella refined (both)	0.148 (0.065)	0.030 (0.100)	70.34	93.98	66.10
Transfers					
CatTSunami	0.157 (0.071)	0.079 (0.177)	66.45	83.82	55.70
Sella refined (any)	0.152 (0.071)	0.094 (0.223)	86.04	83.77	72.08
Sella refined (both)	0.149 (0.068)	0.052 (0.125)	50.49	89.68	45.28

a Sella refined (any) is the case where we take converged Sella results but fall back on converged NEBs when Sella does not converge, and Sella refined (both) is the case where we only keep results where both the NEB and Sella converge.

In addition to the metrics included for all reactions, Table 2 also includes information for each reaction subtype. It should be noted that among desorptions, there are a significant number of calculations that are barrierless (maximum energy along the trajectory is within 0.1 eV of the reactant and product states). In this case, the ML approach was classified as successful if it determined the reaction to be barrierless as well.

**Figure 5:**
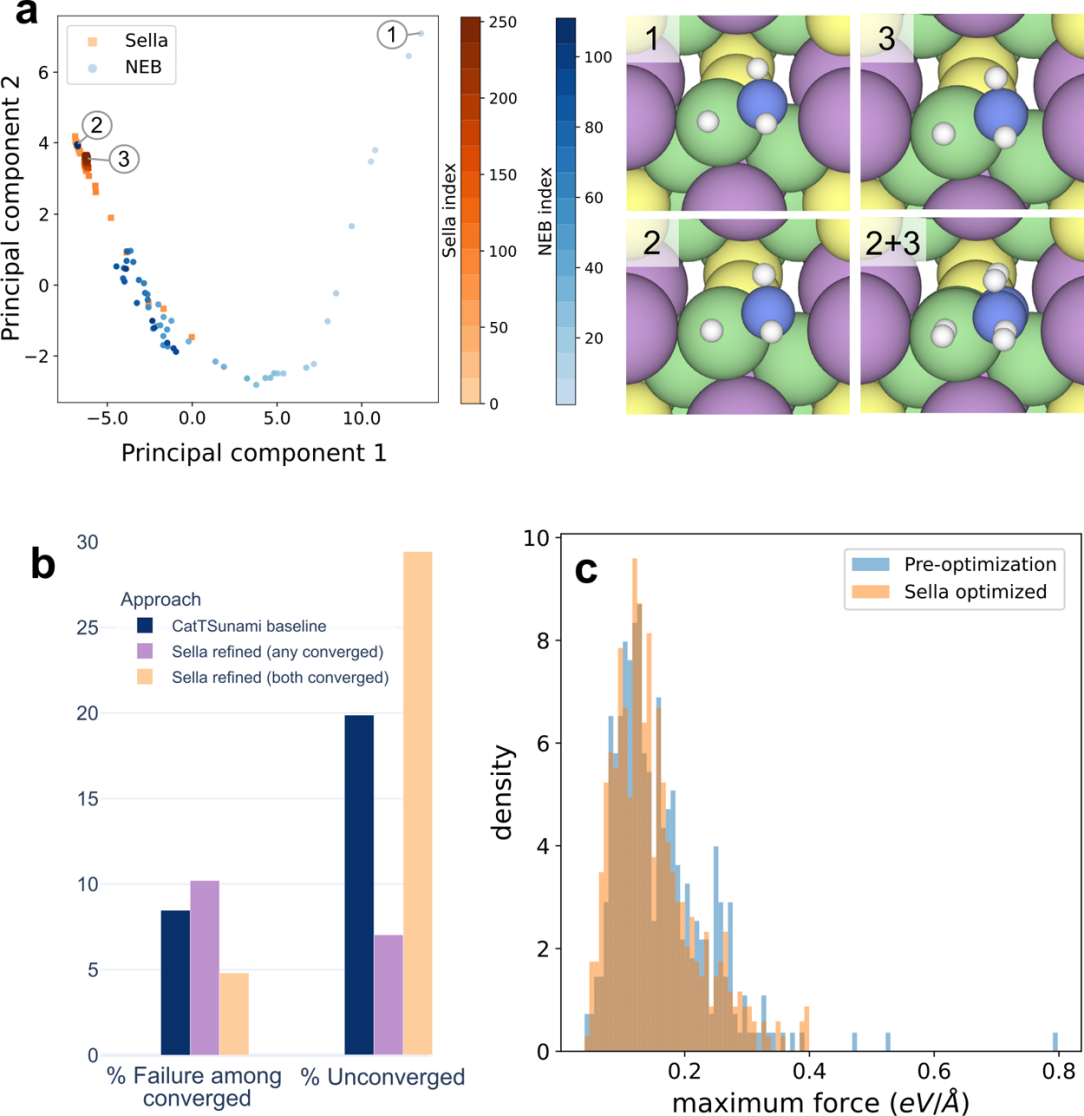
A summary of results for using Sella to perform transition state optimization on OC20NEB systems. (a) A look at the evolution of one system across the NEB trajectory and the Sella optimization trajectory using principal component analysis. The unrelaxed, NEB relaxed, and Sella refined transition states are shown in images 1, 2, and 3, respectively. (b) The % failures among converged calculations and % unconverged through the use of Sella refinement by two different approaches. (c) The distribution of the maximum force (by DFT single point) before and after Sella refinement.

The percentage of systems that are unsuccessful and do not converge are shown in Figure 5b. Using Sella to refine with any converged to optimize for convergence makes a significant difference. It reduces the number of unconverged systems by 65% over the baseline. Using Sella to refine with both converged reduces the number of unsuccessful systems by 41%. A benefit of using the Sella refinement on top of the NEB algorithm can be seen in Figure 5c, where the maximum force distribution is plotted before and after Sella refinement. When the Sella algorithm converges, it tends to remove higher force outliers from the distribution, resulting in more structures which are reasonably close to convergence. It also results in a modest overall shift of the data toward lower Fmax values. In Figure 5a we see an example of what this refinement does to the transition state structure. Plotted on the left are the first two principal components of a principal component analysis performed on the latent space of Equiformer V2, according to the approach used by Musielewicz et al.^48^ We observe that the Sella optimization retraces a similar trajectory to the end of the NEB optimization, and hones in on a slightly more relaxed transition state. This results in a subtle difference in positions of the adsorbate atoms, as seen on the right (2 + 3). In practice, combining these transition state optimizations, with MLP driven estimates of Gibbs free energy corrections, promises to enable MLPs to make wholesale predictions of key kinetic parameters.

## Conclusions

In this work, we demonstrate that models pretrained on the OC20 data set^14^ are able to determine numerical energy Hessians for in domain systems with good accuracy. To facilitate the assessment of model performance, we constructed a data set of 4599 numerical revised Perdew–Burke–Ernzerhof (RPBE) DFT Hessians. In our assessment, we considered four different pretrained model architectures, and also considered the opportunity to fine-tune a model to improve performance.

To understand the implications of this auxiliary ability, we considered the usefulness in application of the energy Hessian to Gibbs free energy corrections and transition state optimization. For Gibbs free energy corrections, the top performing model has an MAE that outperforms (0.042 versus 0.058 eV at 300 K) the commonly used practice of calculating the per adsorbate correction on one surface and taking that value to be true for all other surfaces. We also explored the opportunity to utilize pretrained MLPs to treat translational entropy in the Gibbs free energy corrections, going beyond the harmonic approximation. We found that 94% of systems had a translational entropy contribution greater than 0.1 eV at 300 K. This effect, of course, would be more pronounced at higher temperatures. For transition state optimization using the Hessian we utilized Sella^29^ with the MLPs to refine the ML-relaxed transition states from the OC20NEB data set. We found that Sella can be used to increase the convergence rate from 80.2 to 93.0%, a 65% reduction in the number of unconverged systems.

## Supporting Information Available

DFT settings, Sella hyperparameters, model performances, mean frequencies per adsorbate, comparison of numerical vs automatic differentiation, and CPES validation (PDF)
