# Supplementary material for: Accessing Numerical Energy Hessians with Graph Neural Network Potentials and Their Application in Heterogeneous Catalysis
Source: J Phys Chem C Nanomater Interfaces. 2025 Feb 10;129(7):3510–21. doi: 10.1021/acs.jpcc.4c07477 (PMC11849433; doi:10.1021/acs.jpcc.4c07477)
Supplement: Supplementary file 1 — jp4c07477_si_001.pdf [file jp4c07477_si_001.pdf]

Supporting Information: Accessing Numerical Energy Hessians with Graph Neural Network Potentials and Their Application in Heterogeneous Catalysis

Brook Wander,[†,‡] Joseph Musielewicz,[†,‡] Raffaele Cheula,[¶,‡] and John R. Kitchin[*,‡]

†*These authors contributed equally to this work.*

‡*Department of Chemical Engineering, Carnegie Mellon University, Pittsburgh, PA 15213, USA*

¶*Department of Physics and Astronomy, Aarhus University, Aarhus, 8000, Denmark*

E-mail: jkitchin@andrew.cmu.edu

DFT settings

DFT calculations were performed with the Vienna Ab initio Simulation Package (VASP)[S1–S4] with periodic boundary conditions and the projector augmented wave (PAW) pseudopotentials.[S4,S5] The external electrons were expanded in plane waves with kinetic energy cut-offs of 350 eV. Exchange and correlation effects were taken into account via the generalized gradient approximation and the revised Perdew-Burke-Ernzerhof (RPBE)[S6,S7] functional, because of its improved description of the energetics of atomic and molecular bonding to surfaces.[S8] Bulk and surface calculations were performed considering a K-point mesh for the Brillouin zone derived from the unit cell parameters as an on-the-spot method, employing the Monkhorst-Pack grid.[S9]

Sella hyperparameters

Table S1: The optimized Sella hyperparameters, for application to OC20-like systems using the Equiformer V2 153M parameter model.

Parameter	Value	Description
γ	0	Convergence criterion for iterative diagonalization.
η	7.0E-4	Finite difference step size (Å).
δ_0	4.8E-2	Initial trust radius (Å).
ρ_{inc}	1.035	Threshold for increasing trust radius.
ρ_{dec}	5.0	Threshold for decreasing trust radius.
σ_{inc}	1.15	Trust radius increase factor.
σ_{dec}	0.65	Trust radius decrease factor.
method	P-RFO	Choice of optimization algorithm.

Per model Hessian performance

Local Minimum MAEs

Method	Eigenvalues $eV/Å^2$	All Frequencies cm^{-1}	LI/SR Frequencies cm^{-1}
Fine tuned EQ2	0.513	29.369	46.446
EQ2 153M	1.485	58.195	63.588
EQ2 31M	2.155	80.027	79.687
GemNet T	2.580	101.830	95.677
PaiNN	4.495	165.483	140.570
SchNet	5.779	193.191	115.808

Transition State MAEs

Method	Eigenvalues $eV/Å^2$	All Frequencies cm^{-1}	LI/SR Frequencies cm^{-1}
Fine tuned EQ2	1.191	74.549	202.010
EQ2 153M	1.797	88.067	102.550
GemNet T	1.950	96.581	109.430
EQ2 31M	2.785	136.198	216.724
SchNet	3.287	163.642	238.127
PaiNN	4.139	217.474	264.510

Vibrational correction distributions

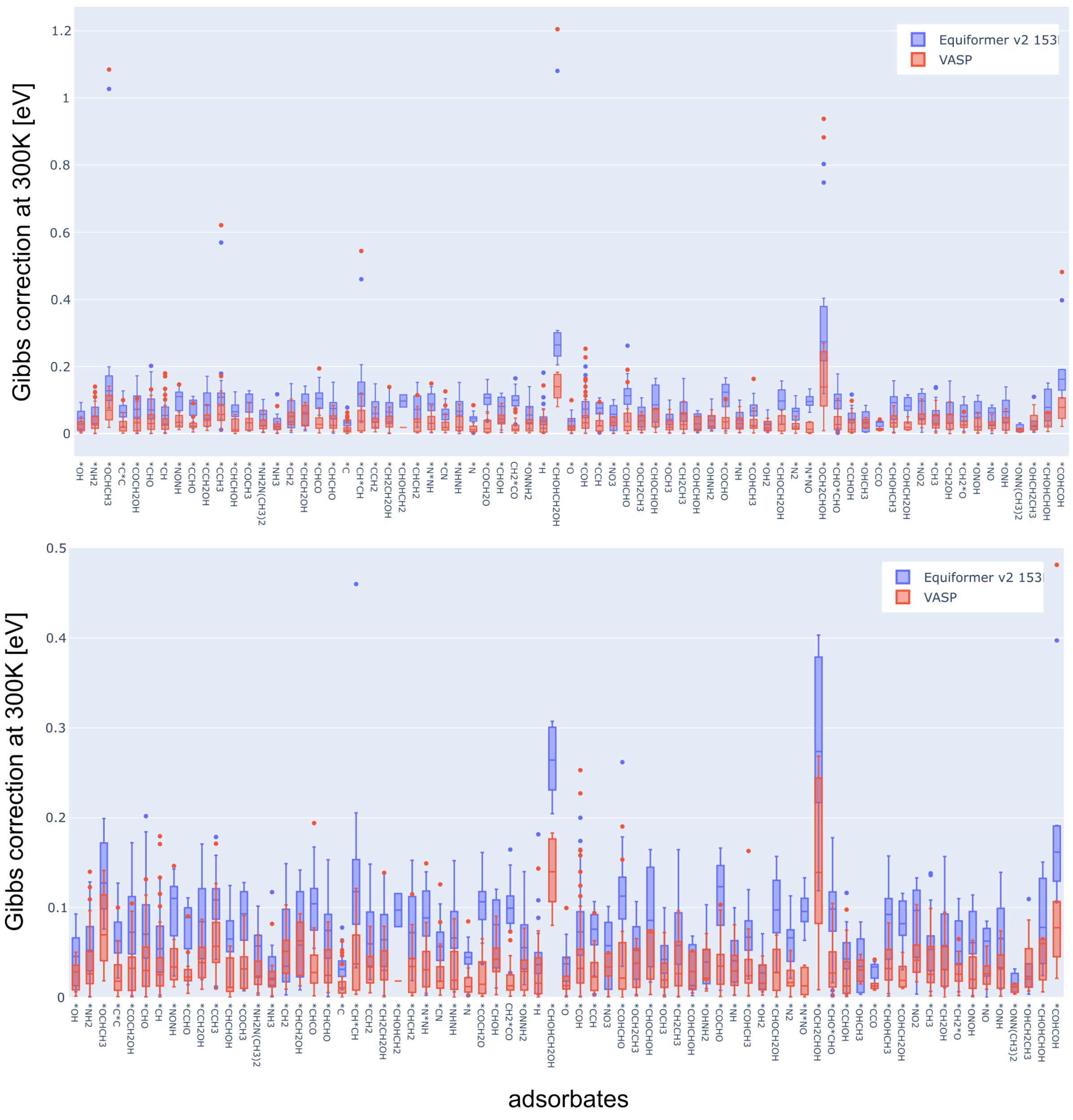

Figure S1: The distribution of Gibbs corrections per adsorbate at 300 K.

OC20Dense single point parities

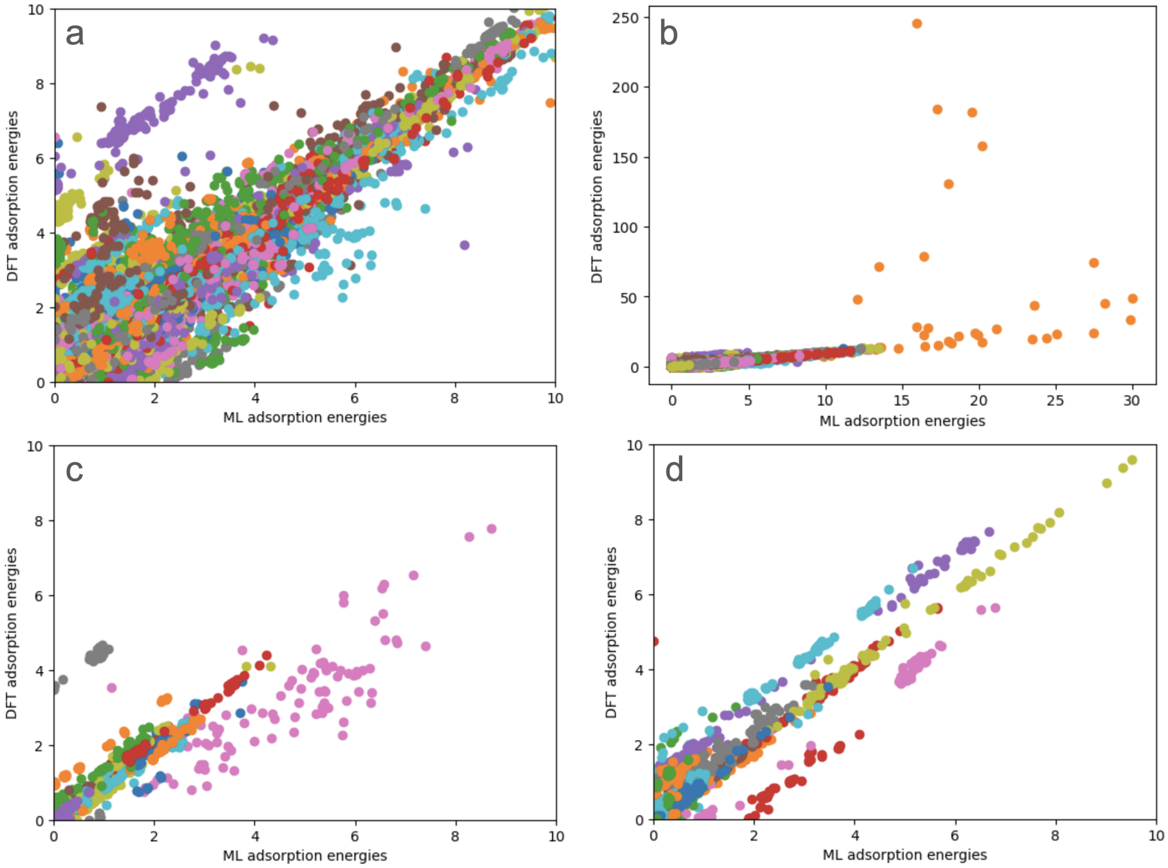

Figure S2: A plot of the relative adsorption energy (relative to the minimum energy) for DFT single points (y-axis) and GemNet-oc ML energies (x-axis) for (a) all OC20Dense systems, (b) all OC20Dense systems with the plot constrained from 0 to 10 eV, and (c,d) A random subselection of systems so aritfacts may be seen in more detail. Each color is an adsorbate + surface combination, though for the plots that show all data, colors are not unique to a single system.

Mean frequencies per adsorbate

adsorbate	mean frequencies
*OH	[108, 180, 276, 447, 718, 3623]
*NH2	[133, 236, 364, 483, 637, 720, 1488, 3273, 3384]
*OCHCH3	[26, 69, 107, 153, 176, 224, 279, 473, 710, 841, 954, 1029, 1172, 1272, 1321, 1366, 1457, 2717, 2797, 2850, 2909]
*C*C	[225, 292, 357, 419, 567, 1490]
*COCH2OH	[35, 75, 103, 143, 190, 259, 326, 408, 490, 570, 866, 956, 1033, 1140, 1234, 1305, 1372, 1496, 2875, 2977, 3476]
*CHO	[114, 185, 251, 332, 472, 659, 1108, 1325, 2694]
*CH	[244, 367, 494, 634, 771, 2911]
*NONH	[45, 111, 157, 217, 288, 340, 619, 745, 1001, 1230, 1402, 3194]
*CCHO	[72, 145, 199, 256, 314, 417, 665, 860, 1107, 1261, 1428, 2842]
*CCH2OH	[40, 94, 167, 214, 280, 345, 436, 585, 830, 939, 1041, 1130, 1220, 1310, 1422, 2784, 2898, 3585]
*CCH3	[55, 113, 179, 271, 324, 477, 881, 933, 1016, 1272, 1362, 1375, 2755, 2819, 2861]
*CHCHOH	[43, 77, 120, 163, 290, 401, 562, 630, 710, 890, 992, 1130, 1264, 1319, 1507, 2947, 3058, 3316]
*COCH3	[28, 93, 124, 159, 235, 273, 402, 556, 872, 940, 1044, 1263, 1361, 1404, 1532, 2830, 2988, 3031]
*NH2N(CH3)2	[10, 32, 58, 85, 107, 163, 244, 270, 348, 412, 473, 540, 800, 971, 1011, 1072, 1090, 1121, 1199, 1235, 1301, 1388, 1413, 1425, 1433, 1444, 1454, 1570, 2844, 2861, 2967, 2982, 3025, 3036, 3143, 3300]
*NH3	[61, 121, 168, 285, 516, 546, 1175, 1571, 1586, 3143, 3359, 3402]
*CH2	[123, 277, 385, 487, 675, 765, 1315, 2820, 2959]
*CHCH2OH	[28, 92, 148, 185, 290, 386, 468, 583, 728, 896, 944, 1067, 1146, 1205, 1297, 1350, 1417, 2828, 2930, 2983, 3495]
*CHCO	[74, 122, 187, 236, 332, 411, 535, 641, 887, 1105, 1682, 2899]
*CHCHO	[70, 112, 169, 242, 315, 387, 635, 744, 901, 1015, 1192, 1295, 1458, 2842, 2979]
*C	[290, 413, 634]
*CH*CH	[144, 238, 273, 395, 491, 586, 799, 920, 1096, 1297, 2812, 2922]

*CCH2	[101, 175, 267, 325, 414, 497, 827, 946, 1285, 1488, 2850, 3022]
*CH2CH2OH	[21, 70, 104, 145, 220, 327, 397, 496, 673, 857, 923, 1010, 1088, 1151, 1224, 1288, 1359, 1390, 1442, 2862, 2920, 2955, 3004, 3487]
*CHOHCH2	[96, 142, 191, 243, 339, 409, 468, 542, 709, 882, 1010, 1054, 1077, 1131, 1211, 1354, 1379, 2940, 2973, 3044, 3548]
*CHCH2	[110, 173, 244, 319, 437, 525, 769, 892, 974, 1186, 1284, 1466, 2685, 2923, 3032]
*N*NH	[143, 231, 289, 388, 458, 599, 1031, 1294, 3184]
*CN	[127, 209, 315, 358, 438, 1699]
*NHNH	[96, 151, 222, 293, 376, 480, 802, 1037, 1256, 1389, 3185, 3248]
*N	[228, 351, 612]
*COCH2O	[59, 107, 155, 198, 278, 339, 401, 503, 547, 832, 957, 1032, 1168, 1257, 1342, 1471, 2844, 2921]
*CHOH	[64, 143, 218, 305, 435, 501, 842, 1056, 1194, 1339, 2754, 3359]
CH2*CO	[91, 151, 202, 269, 332, 428, 568, 659, 776, 926, 1068, 1307, 1485, 2971, 3100]
*ONNH2	[16, 38, 73, 116, 145, 182, 376, 584, 692, 1020, 1189, 1326, 1527, 3164, 3475]
*H	[455, 714, 1468]
*CHOHCH2OH	[45, 65, 93, 139, 178, 217, 277, 358, 409, 494, 592, 768, 821, 921, 959, 1016, 1072, 1137, 1219, 1270, 1307, 1364, 2699, 2759, 2789, 3202, 3301]
*O	[179, 288, 518]
*COH	[76, 162, 248, 312, 401, 511, 996, 1183, 3352]
*CCH	[112, 212, 291, 355, 440, 615, 815, 1534, 3130]
*NO3	[34, 60, 79, 137, 187, 243, 603, 623, 719, 890, 1108, 1277]
*COHCHO	[55, 113, 152, 197, 235, 294, 362, 415, 480, 595, 810, 965, 1068, 1183, 1295, 1443, 2907, 3495]
*OCH2CH3	[13, 43, 79, 127, 179, 232, 311, 477, 786, 876, 1020, 1076, 1122, 1254, 1332, 1356, 1431, 1438, 1455, 2884, 2917, 2944, 3013, 3028]
*CHOCHOH	[52, 93, 136, 175, 283, 319, 342, 483, 579, 662, 781, 907, 1001, 1097, 1184, 1264, 1321, 1429, 2922, 3084, 3514]
*OCH3	[42, 81, 118, 169, 244, 373, 1027, 1124, 1141, 1414, 1433, 1440, 2887, 2954, 2973]
*CH2CH3	[36, 88, 134, 204, 243, 400, 577, 899, 933, 1003, 1146, 1213, 1337, 1383, 1430, 1448, 2773, 2870, 2937, 2980, 3014]
*COHCHOH	[6, 38, 69, 136, 196, 231, 316, 392, 435, 488, 568, 800, 996, 1093, 1187, 1269, 1318, 1549, 3032, 3359, 3530]

*OHNH2	[18, 68, 114, 142, 197, 265, 548, 785, 1132, 1249, 1326, 1578, 3166, 3297, 3437]
*COCHO	[90, 131, 184, 242, 285, 340, 392, 485, 593, 785, 1009, 1148, 1296, 1457, 2972]
*NH	[194, 305, 482, 585, 783, 3331]
*COHCH3	[18, 54, 85, 135, 220, 250, 347, 503, 640, 866, 961, 1031, 1156, 1277, 1339, 1392, 1431, 2798, 2961, 3002, 3329]
*OH2	[52, 115, 178, 284, 470, 549, 1547, 3287, 3538]
*CHOCH2OH	[50, 85, 121, 153, 196, 265, 323, 411, 513, 610, 787, 872, 966, 1024, 1112, 1185, 1259, 1315, 1349, 1457, 2742, 2914, 2985, 3479]
*N2	[143, 201, 319, 360, 398, 1553]
*N*NO	[67, 128, 184, 269, 347, 439, 756, 1114, 1419]
*OCH2CHOH	[29, 71, 115, 158, 191, 234, 281, 342, 390, 478, 645, 792, 875, 945, 1006, 1079, 1128, 1201, 1276, 1359, 2462, 2579, 2782, 3311]
*CHO*CHO	[77, 113, 153, 198, 256, 307, 398, 512, 718, 774, 837, 952, 1118, 1230, 1337, 1450, 2884, 2955]
*CCHOH	[24, 83, 148, 215, 291, 357, 582, 644, 846, 1053, 1197, 1297, 1479, 3016, 3427]
*OHCH3	[14, 55, 91, 138, 162, 248, 537, 957, 1064, 1136, 1290, 1415, 1435, 1449, 2843, 3006, 3049, 3298]
*CCO	[33, 63, 184, 265, 335, 529, 577, 1243, 2044]
*CHOHCH3	[48, 85, 115, 194, 228, 269, 371, 432, 508, 791, 930, 997, 1046, 1119, 1223, 1315, 1354, 1418, 1451, 2833, 2894, 2976, 3011, 3440]
*COHCH2OH	[33, 68, 103, 137, 196, 240, 291, 363, 462, 505, 595, 874, 952, 999, 1083, 1151, 1236, 1296, 1333, 1423, 2887, 2944, 3315, 3504]
*NO2	[94, 134, 187, 258, 302, 401, 621, 864, 1055]
*CH3	[88, 163, 212, 420, 601, 659, 1172, 1359, 1391, 2782, 2937, 2994]
*CH2OH	[34, 88, 136, 240, 354, 491, 642, 896, 1079, 1161, 1284, 1410, 2921, 3016, 3530]
*CH2*O	[85, 144, 200, 295, 376, 520, 908, 1132, 1217, 1445, 2829, 2943]
*ONOH	[28, 76, 116, 144, 181, 225, 468, 599, 831, 1084, 1361, 3440]
*NO	[68, 123, 257, 321, 445, 1377]
*ONH	[91, 148, 200, 278, 364, 531, 963, 1325, 3157]
*ONN(CH3)2	[9, 22, 36, 60, 77, 106, 127, 150, 212, 305, 349, 403, 665, 824, 1000, 1028, 1065, 1102, 1185, 1272, 1304, 1384, 1409, 1421, 1431, 1446, 1464, 2932, 2945, 2998, 3015, 3060, 3069]
*OHCH2CH3	[8, 22, 37, 82, 96, 150, 227, 336, 460, 798, 851, 1007, 1038, 1108, 1231, 1285, 1356, 1379, 1438, 1445, 1462, 2912, 2942, 2982, 3015, 3028, 3519]

*CHOHCHOH	[17, 69, 106, 139, 185, 232, 298, 352, 447, 553, 634, 734, 876, 1026, 1083, 1154, 1251, 1275, 1329, 1523, 2921, 3058, 3245, 3524]
*COHCOH	[52, 81, 119, 194, 226, 247, 286, 359, 466, 534, 701, 889, 1023, 1137, 1237, 1344, 3176, 3345]

Numerical Differentiation vs Analytical Differentiation

Unlike VASP, the outputs of graph neural networks (GNN) potentials can be differentiated analytically. This allows the the Hessian matrix to be computed from the first derivative of the predictions analytically, instead of by finite differences. We implemented both approaches, and found the predicted Hessian to achieve close agreement ($R^2 = 0.995$) between the two approaches for a specific finite difference displacement size of 1.0E-3 Å. This differs slightly from the displacement size of 7.5E-3 actually used for the VASP Hessian dataset, which introduced some noisy error when computing the agreement between the VASP Hessian and the analytical GNN Hessian. This error was not produced when using the numerical GNN Hessian produced by finite differences with the same displacement size as VASP. Analyzing the runtimes for both the numerical and analytical approaches of obtaining potential energy Hessian with the highest performing GNN architectures, we observed that our implementation of automatic differentiation (using the autograd package) required significantly longer compute times to resolve. On average, for Equiformer V2, our implementation of the analytical Hessian required approximately six times as many node hours as performing all the inference calls for the finite differences method. This result should depend on the number of adsorbate atoms being displaced however, since the finite differences approach adds six displacement dimensions per atom. This result is surprising, but after troubleshooting we were unable to determine an obvious culprit, therefore we elected to use numerical Hessians derived from finite differences for all GNN potential experiments in this work. If a more efficient approach to calculating the analytical Hessian on GNN potentials could be implemented (particularly for Equiformer V2), with compute time on the order of a single inference call, then our results suggest this would be a favorable approach to computing the Hessian for GNN potentials. As it stands, the compute costs of performing inference on GNN potentials is substantially lower than resolving VASP calculations, therefore we are satisfied that the numerical approach is currently worth using.

Validation of CPES with ML

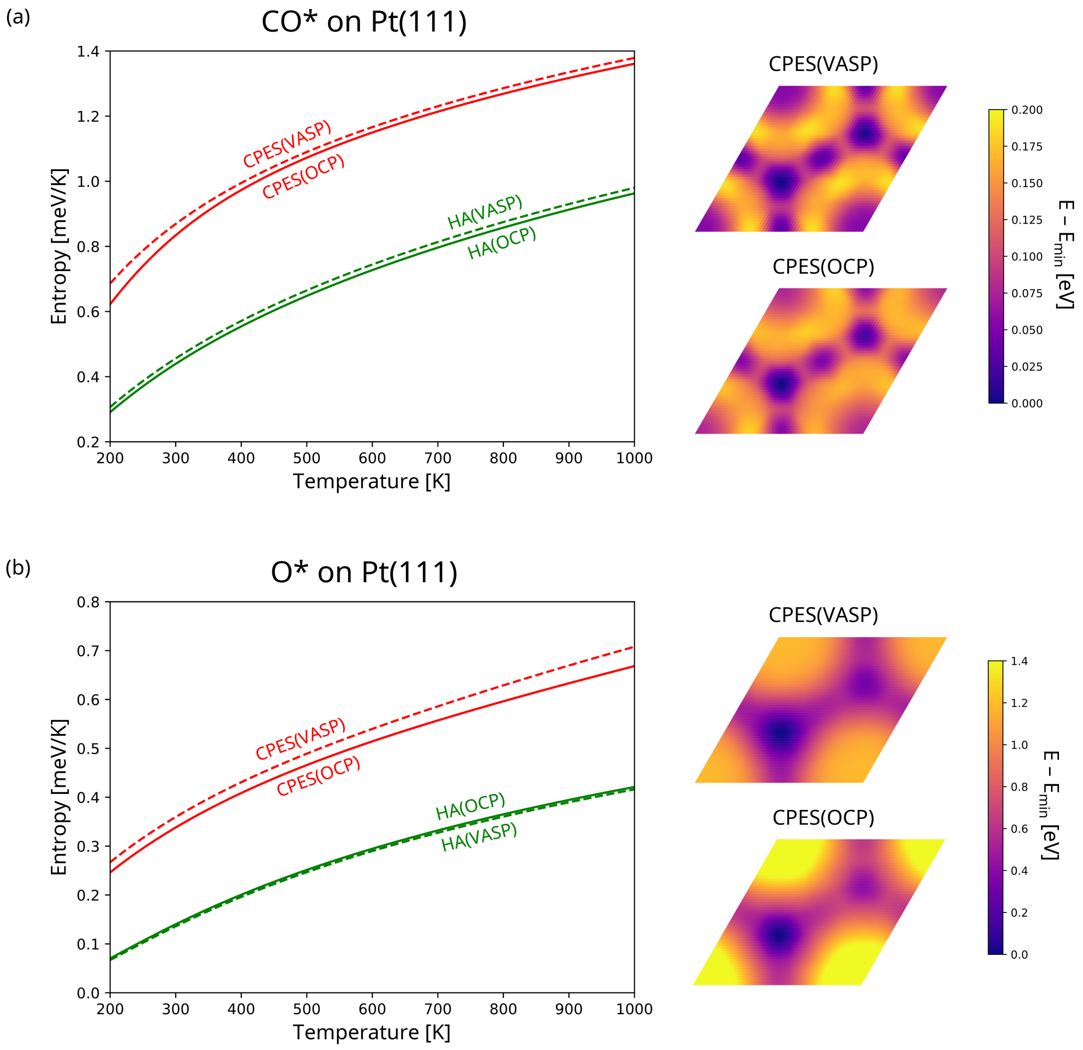

Figure S3: Plots of entropy vs temperature for CO* (a) and O* (b) on Pt(111), obtained with the harmonic approximation (HA) and the complete potential energy sampling (CPES) methods, and using energies calculated with VASP or the pretrained OCP ML potential. The 2D plots of potential energy surfaces are also shown in the figure.

Comparing the entropy from HA and CPES

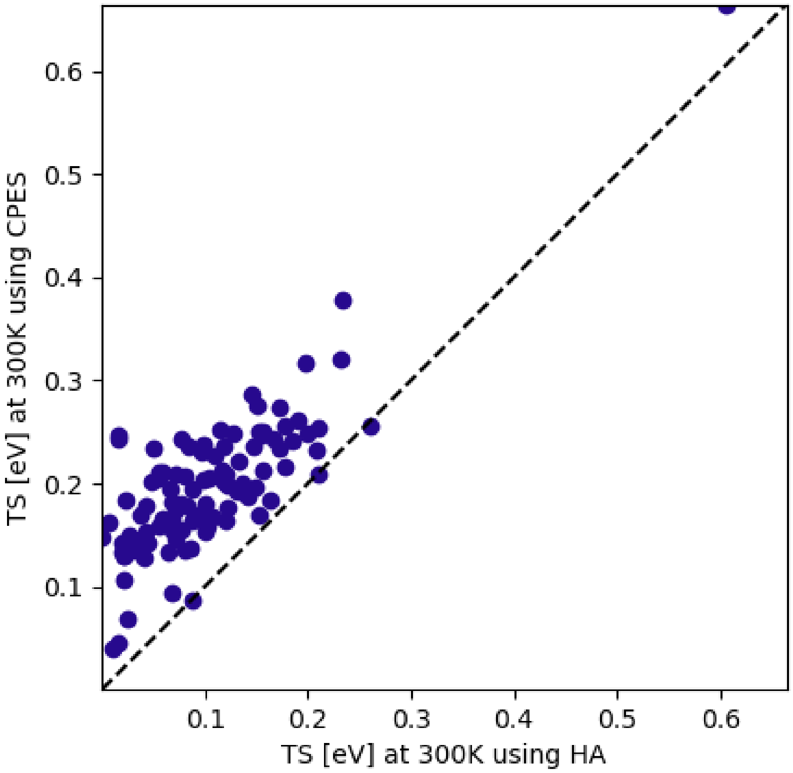

Figure S4: A parity between the TS term that would be determined using the harmonic approximation (HA) versus one determined by complete potential energy sampling (CPES).

References

(S1) Kresse, G.; Hafner, J. Ab initio molecular-dynamics simulation of the liquid-metal–amorphous-semiconductor transition in germanium. *Physical Review B* **1994**, *49*, 14251.

(S2) Kresse, G.; Furthmüller, J. Efficiency of ab-initio total energy calculations for metals and semiconductors using a plane-wave basis set. *Computational materials science* **1996**, *6*, 15–50.

(S3) Kresse, G.; Furthmüller, J. Efficient iterative schemes for ab initio total-energy calculations using a plane-wave basis set. *Physical review B* **1996**, *54*, 11169.

(S4) Kresse, G.; Joubert, D. From ultrasoft pseudopotentials to the projector augmented-wave method. *Physical review b* **1999**, *59*, 1758.

(S5) Blöchl, P. E. Projector augmented-wave method. *Physical review B* **1994**, *50*, 17953.

(S6) Perdew, J. P.; Burke, K.; Ernzerhof, M. Generalized gradient approximation made simple. *Physical review letters* **1996**, *77*, 3865.

(S7) Zhang, Y.; Yang, W. Comment on "Generalized gradient approximation made simple". *Physical Review Letters* **1998**, *80*, 890.

(S8) Hammer, B.; Hansen, L. B.; Nørskov, J. K. Improved adsorption energetics within density-functional theory using revised Perdew-Burke-Ernzerhof functionals. *Physical review B* **1999**, *59*, 7413.

(S9) Monkhorst, H. J.; Pack, J. D. Special points for Brillouin-zone integrations. *Physical review B* **1976**, *13*, 5188.
